# Cholesterol Stationary Phase in the Separation and Identification of siRNA Impurities by Two-Dimensional Liquid Chromatography-Mass Spectrometry

**DOI:** 10.3390/ijms232314960

**Published:** 2022-11-29

**Authors:** Sylwia Studzińska, Feiyang Li, Michał Szumski, Bogusław Buszewski, Michael Lämmerhofer

**Affiliations:** 1 Chair of Environmental Chemistry and Bioanalytics, Faculty of Chemistry, Nicolaus Copernicus University in Toruń, 7 Gagarin Str., 87-100 Toruń, Poland; 2 Institute of Pharmaceutical Sciences, Pharmaceutical (Bio-)Analysis, University of Tübingen, Auf der Morgenstelle 8, 72076 Tübingen, Germany; 3 Centre for Modern Interdisciplinary Technologies, Nicolaus Copernicus University in Toruń, 4 Wilenska St., 87-100 Toruń, Poland

**Keywords:** oligonucleotide, impurities, stationary phase, separation, two-dimensional liquid chromatography, mass spectrometry

## Abstract

The aim of this research was to develop a simple and efficient ion-pair reagent-free chromatographic method for the separation and qualitative determination of oligonucleotide impurities, exemplified by synthesis of raw products of the two single strands of patisiran siRNA. The stationary phases with mixed hydrophobic/hydrophilic properties (cholesterol and alkylamide) were firstly used for this purpose with reversed-phased high-performance liquid chromatography. Several different chromatographic parameters were tested for their impact on impurities separation: type, concentration, pH of salt, as well as organic solvent type in the mobile phase. The pH was the most influential factor on the separation and signal intensities in mass spectrometry detection. Finally, the optimized method included the application of cholesterol stationary phase, with mobile phase containing 20 mM ammonium formate (pH 6.5) and methanol. It allowed good separation and the identification of most impurities within 25 min. Since not all closely related impurities could be fully resolved from the main peak in this oligonucleotide impurity profiling, two-dimensional liquid chromatography was used for peak purity determination of the target oligonucleotides. The Ethylene Bridged Hybrid (BEH) Amide column in hydrophilic interaction liquid chromatography was applied in the second dimension, allowing additional separation of three closely related impurities.

## 1. Introduction

In manufacturing a drug and ensuring its proper therapeutic activity, one of the most important factors is the purity of the active substance. Impurities must be firstly qualitatively determined so that a purification method could be successfully developed. This stage of drug development is often difficult, particularly for oligonucleotides (OGNs), which are increasingly used to treat a wide range of diseases [1,2,3]. Therapies involve the introduction of synthetic, usually chemically modified OGNs into the cell [1,2]. Various OGNs with different mechanisms of action are currently being used: antisense OGNs, micro-RNAs, aptamers, and small interfering RNAs [4,5,6]. Their impurities can be classified into several groups: products of modification at a single phosphodiester linkage, sugar or base residue, products of deletion of a single nucleotide (N − 1, N − 2), or incorporation of a single nucleotide repeat (N + 1) and other longmers [7,8]. The difficulties associated with characterizing these impurities are their large molecular weights and closely related structure [3,8]. For this reason, their complete structural elucidation is challenging, and the development of improved analytical methods should be pursued to enable their separation from the parent OGN and from each other [3,7,8].

Historically, the first separation technique used for this purpose was anion exchange chromatography (AEC). However, it has been used less frequently recently, due to its incompatibility with mass spectrometry (MS) [3,9]. In some cases, capillary gel electrophoresis was also applied [10]. The most popular technique for achieving length-based separations is high-performance liquid chromatography, especially in ion-pair mode (IP RP HPLC) [3,11,12,13,14,15,16]. Its combination with electrospray ionization mass spectrometry (ESI-MS) makes it generally suitable for the analysis of impurities, due to, e.g., the high mass spectral response [11,12,13]. Consequently, IP RP HPLC ESI-MS has become a method of first choice for the analysis of impurities, although it often fails to separate impurities such as N − 1 or N + 1 that co-elute with the parent OGN [13]. Moreover, even though ion pair reagents are used in the mobile phase, the separation efficiency of IP RP HPLC for a complex mixture of impurities that elute before and after the main OGN is often limited, tedious, and time-consuming [13,14]. The application of methods for automated impurities determination or mobile phases containing small alkylamines may be useful, as shown by Roussis et al. [13,14,15,16]. However, even for an optimized IP RP HPLC method, the complete separation of positional isomers and isobaric species for example still remains very challenging [15,16]. Moreover, the utilization of alkylamines in the mobile phase causes ionization suppression, ion source contamination, and MS memory effect [17]. For these reasons, additional methods are continuously developed, such as new methods based on the application of various stationary and mobile phases (e.g., hydrophilic interaction liquid chromatography—HILIC, reversed-phase liquid chromatography—RP HPLC, or mixed mode liquid chromatography) or combining of two different liquid chromatography modes in two-dimensional liquid chromatography (2D-LC) [18,19,20,21,22,23,24,25,26]. Each of these attempts usually improves separation, MS sensitivity, or simplifies the method compared to IP RP HPLC. Comprehensive RP HPLC × IP RP HPLC, AEC × IP RP HPLC provides orthogonal separation of some isobaric impurities [24]. Moreover, a multiple heart-cutting AEC—HILIC, IP RP HPLC—HILIC, AEC RP HPLC, or mixed mode RP/AEC—RP HPLC allow online desalting, the separation of additional impurities, and the identification of impurities by MS in the absence of ion-pairing reagents, respectively [22,23,26]. On the other hand, 2D-LC has its limitations, namely dilution problems, as well as the necessity of high peak capacity in the first dimension (^1^D) so that the majority of components can be separated in the ^1^D [24,26]. Interestingly, mixed-mode stationary phases with anion-exchange and hydrophobic moieties at the surface exhibited good separation potential for structurally related OGNs [18,27]. Nevertheless, eluents containing sufficiently high concentrations of counterions (usually MS-incompatible) are required in order to elute OGNs [18,24]. Based on this short literature review, in our opinion, there is still a gap to fill with new methods allowing OGN impurities separation and identification.

The main goal of the present study was to improve the resolution via the application of mixed-mode stationary phases in RP HPLC for the separation of patisiran analogue (as model siRNA OGN) from its impurities and their identification by quadrupole time-of-flight mass spectrometry (Q-TOF-MS). This made it possible to test whether the use of such a simple chromatographic system (stationary phases of mixed properties and MS-compatible mobile phases) could be useful in the analysis of OGN impurities. Cholesterol and alkylamide columns were evaluated for their selectivity under reversed phase conditions to elucidate their potential for OGN studies. Moreover, the retention orthogonality between cholesterol and amide stationary phases (in HILIC mode) was also tested in order to verify the possibility of 2D-LC application to improve separation power.

## 2. Results and Discussion

### 2.1. Retention and Separation Studies for CHOL and AP Columns

**Figure 1:**
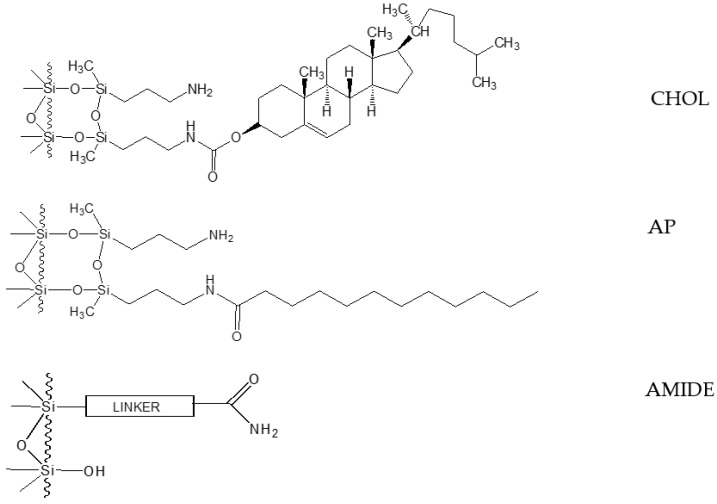
Schematic structure of stationary phases used in the present study.

Two different stationary phases were chosen for the preliminary study. Our previous work has shown that the use of a reversed-phase system for the determination of OGN impurities is successful if stationary phases with mixed hydrophobic/hydrophilic properties are used. For these reasons, CHOL and AP columns were tested in the present study. Both stationary phases have previously been used for the separation of OGN mixtures, but only in ion pair mode [27,28]. The aim herein was to test their suitability in the much simpler reversed-phase mode, since both polar (residual silanols, amino, amide groups) and non-polar groups (cholesterol molecule or alkyl chain) are localized in their structure, so they appear to be good candidates for the separation and analysis of OGN impurities (Figure 1).

**Figure 2:**
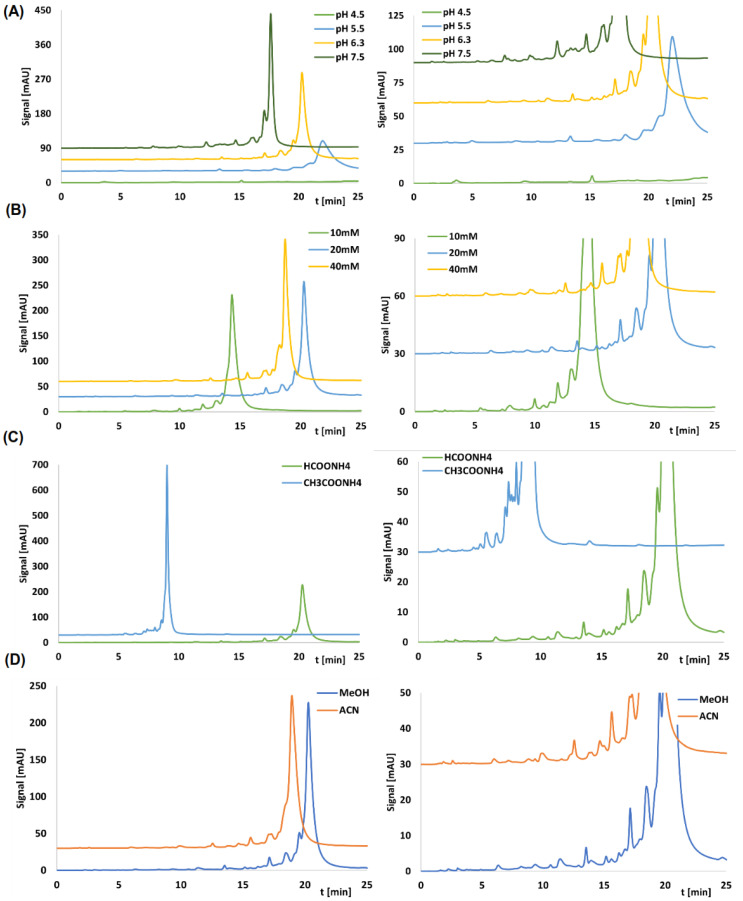
Exemplary chromatograms (left side) with the enlarged view (right side) for sense strand of patisiran analogue for CHOL stationary phase obtained for: (**A**) different pH values (for 20 mM ammonium formate); (**B**) different salt concentration (for ammonium formate); (**C**) two different salts (both of them were 20 mM solutions); (**D**) two different organic solvents. Experimental conditions: MPA: salt solution; MPB: MeOH/salt solution 9:1 (*v*/*v*); gradient elution: 10–45% MPB in 20 min (20 min re-equilibration); column temperature, 40 °C; autosampler temperature, 10 °C; flow rate, 0.3 mL/min; injection volume, 8 µL.

Retention studies were conducted under gradient elution conditions, with the elution program remaining constant regardless of the column or chromatographic parameter being tested. During the preliminary experiments, we tested the influence of various parameters on impurity separation and retention: pH, salt concentration, type of salt, and type of organic solvent. Table 1 summarizes the retention factor values (*k*) for the main compound of sense and antisense strands for different parameters, whereas Figure 2 shows selected chromatograms for CHOL stationary phase. The other most important chromatograms are collected in the Supplementary Materials (Figure 2 and Figures S1–S3).

Irrespective of the chromatographic column used, the trends were analogous. With higher pH, the retention of the main compounds decreases (Figure 2A, Figures S1A and S2A, Table 1). It is the effect of protonation of the residual aminopropyl groups that leads to enhanced retention of tested compounds due to electrostatic interactions with negatively charged OGNs. The use of ammonium formate with a pH 4.5 resulted in a lack of elution of the OGNs under the conditions of the gradient used (they were eluted from the column during the washing step). As pH decreased, not only the retention of the main compounds (sense or antisense strands) increased, but also the retention of impurities (Figure 2A, Figures S1A and S2A). This should have resulted in a better separation of the structurally related impurities for pH 5.5, but the width of the peaks also increased with decreasing pH (due to an increased contribution of hydrogen bonding and electrostatic interactions in the retention mechanism). As a result, peak intensities decreased with pH 5.5, which had a negative impact on detection. Consequently, pH 6.3 and 7.5 were selected for further testing.

With regard to the influence of the salt concentration (for ammonium formate, pH 6.3), the observed effects were the same for CHOL and AP columns (Figure 2B, Figures S1B and S2B). When raising the concentration from 10 to 20 mM, the *k* values increased, but a further increase in concentration to 40 mM resulted in a reduction of retention (Table 1). These effects can be explained by a superposition of two retention principles, viz. reversed-phase LC (an increase of OGN retention with increasing salt concentration) and ion chromatography (reduction of retention with increasing concentration). It can be assumed that in the case of tested stationary phases, the dominating retention mechanism may change depending on the chromatographic conditions used, e.g., for low pH of salt or high salt concentration, ion exchange becomes predominant. The best separation of impurities was achieved with 20 and 40 mM ammonium formate (Figure 2B, Figures S1B and S2B), due to the greater retention. For further studies, 20 mM was selected due to the intended identification of impurities by Q-TOF-MS, for which a high concentration of 40 mM would lead to ion suppression and contamination of the ion source.

Ammonium acetate has a higher elution strength of OGNs compared to ammonium formate (for the same concentration of both salts) for CHOL and AP. This effect is greater for the CHOL, for which an almost twofold reduction of *k* values was observed (Table 1). In both cases, better separation of impurities was obtained for ammonium formate (Figure 2C and Figure S1C).

The final parameter tested in this research was the type of organic solvent. As expected, OGNs and their impurities were eluted from the column more rapidly with acetonitrile (Figure 2D and Figure S1D). However, this resulted in a lower resolution due to insufficient retention, which is why methanol was finally chosen.

Comparing the results obtained for CHOL and AP (Figure S3), it should be noted that in the case of AP, the retention of the studied OGNs and their impurities was always lower. This is most probably the result of the differences in the structure of both stationary phases, as according to the results obtained previously in our group, their hydrophobicity is almost the same [29] (Figure 1). The CHOL phase has a higher carbon content (9.97%) than AP (6.52%) because the ligand molecule is larger (more bulky) than C12 of the AP ligand (which is straight, but can collapse at high water content). However, the surface density of the CHOL ligands is lower (2.61 µmol/m^2^). With a lower surface density of CHOL, the separated molecules have a chance to enter between CHOL ligands, whereas on more densely distributed C12 (3.49 µmol/m^2^), they may slide on their top instead [29]. So, together with good interaction with the CHOL molecules, some interaction with aminopropyl groups or so called “hydrolytic pillow” is possible [30]. Taking this into account, it is very likely that better separation on the CHOL phase is possible due to the steric effect and electrostatic interactions with residual amino groups.

### 2.2. Impurities Analysis with the Use of Q-TOF-MS

In the next stage of the study, Q-TOF-MS was used to identify impurities in both OGNs. In line with previous results, we tested two different pH values of ammonium formate. These were chosen because of the satisfactory separation of the impurities, but it was necessary to additionally check how the pH of the salt in the mobile phase affects the sensitivity, which is an equally important parameter in the determination of impurities. For this reason, we compared peak areas at extracted ion chromatograms (EIC) for several selected ions (assuming that tendencies for other ions were the same). The results are summarized in Table 2. Surprisingly, larger peak areas were obtained for the mobile phase, which included a salt with a lower pH. The OGNs are negatively charged in the whole range of pH used during the present study (due to the pK_a_ values of phosphate groups). Despite the fact that nucleobases can also contribute to the overall charge of OGN (protonation), the phosphate backbone will contribute to the greatest extent. Consequently, it will be also the most influential factor during the electrospray ionization of OGNs. It has been shown that usually for high pH values of the mobile phase, their ionisation is more efficient; hence, higher peak areas and sensitivity in mass spectrometry, especially in ion pair chromatography, are observed [31,32]. However, our results for the two tested OGNs do not entirely confirm these effects, as we have obtained higher sensitivity for lower pH.

Despite the differences in intensity, exactly the same impurities were identified for both mobile phases. Thus, in this case, the higher peak areas for pH 6.3 did not lead to the identification of more impurities (due to the higher sensitivity). With regard to relative abundance of the different charge states, no differences were observed here either. For both pH 6.3 and 7.5, the same charge states (either −2 or −3) were the most abundant ones (Figure S4).

The low concentrations of most of the impurities present in the sample caused the presence of only one charge state at the full scan spectra (e.g., −1 for *m/z* 1011.149, 865.173 ions, and −2 for *m/z* 915.668, 1075.689 ions) (Figure S5). In the case of the main compound (sense and antisense strands of patisiran analogue), for which the concentration was the highest, three different charge states were observed, whereas for compounds with intermediate concentrations, there were two charge states, typically −2 and −3 or −3 and −4 (e.g., *m/z* 823.469 (−3) and 1235.711 (−2) for 5′ N-13s; *m/z* 1153.181 (−3) and 1733.289 (−2) for 5′N-14s; *m/z* 1489.237 (−3) and 1116.928 (−4) for 5′ N-7s) (Table 3, Figures S5 and S6). For some impurities, the ion with the lower charge state was more abundant, whereas for others, it was the one with the higher charge state (Table 3, Figures S4–S6).

Deconvolution was used during the study to provide zero-charge state mass spectra and accurate masses of OGN impurities. The PeakView software equipped with Bio Tool Kit Plug-in was used, and deconvolution was performed according to the scheme presented in Figure S7. The neutral monoisotopic masses of OGN impurity series in which each OGN had one nucleotide less were calculated. Primarily, the structural assignment of the nucleotide sequence was based on MS1 accuracy between experimental and calculated masses. Secondly, the structural assignment of the nucleotide sequence was confirmed by MS2 spectra. RoboOligo and OPA/OMA developed by Limbach et al. [33] and Schürch et al. [34] were also used for this purpose. Moreover, the annotated sequences were confirmed by the MS/MS spectra which feature characteristic fragment ions of the oligonucleotide impurities. Exemplary fragmentation spectra for several impurities of sense and antisense patisiran were presented in Figures S8 and S9. The most common fragmentation pathway corresponds to the loss of a nucleobase followed by inter-nucleotide backbone cleavage. The ions were assigned to the sequence fragments based on the oligonucleotide MS/MS fragmentation scheme proposed by McCloskey (Figure S10). Oligonucleotides are fragmentated along the phosphate backbone and produce a set of ions containing the 5′ terminus (a, a-B, b, c, d) and 3′ end group (w, x, y) (Figures S8G–I and S9G–I). The number of nucleotides from the end group is presented as the subscript to the letter. The a-B ions orginate from base loss (cleavage of *N*-glycoside bond) in addition to cleavage at the phosphodiester bonds at the 3′ or 5′ end. Representative examples are shown for the sense strand of patisiran (Figure S8G–I). In the case of our study with CID, signals from b, c, y, and w ions were mainly observed in the MS/MS spectra (Figures S8 and S9).

#### 2.2.1. The Chemistry behind the Origin of Impurities

Table 3 presents the most abundant ion of each impurity, its preferential charge state, deconvoluted mass, and annotated sequence, whereas the relative proportion for each impurity appointed as peak area and relative peak area for both MS and UV were collected in Table S1 Typically, impurities are formed as N-shortmers, but base mismatches are also possible. Additionally, at the 3′ end of the sequence the hydroxy (OH), phosphate (PO) or 2′,3′-cyclic phosphate (cyc) group may be present. Twenty-eight (28) impurities were annotated for the antisense strand of patisiran analogue, and twenty-two (22) for the sense strand. In both cases, a greater number of 5′-shortmers was found compared to 3′ ones (Table 3). In our study, we have only observed impurities formed as N-shortmer sequences of the main compound. Consequently, the failure sequences lacked various numbers of nucleotides from the 5′ and 3′ end of the target patisiran single nucleotide strands (Table 3). Similar types of impurities were already reported in the scientific literature [9,14]. The incomplete coupling (so-called coupling failures) of nucleotides during the synthesis leads to a number of impurities, which were presented in Table 3, e.g., coupling failure sequence formed in this way missing the last nine nucleotides from its 5′ end is described as the 5′ (N-9)as (for antisense strand of patisiran) or 5′ (N-9)s (for sense strand of patisiran analogue).

The results in Table S1 contain the relative and absolute signal values detected for each impurity and main compound. The absolute signal of the detected impurities is used only for relative comparisons, since direct use of these data for absolute quantification of impurities is not appropriate. The greatest relative and absolute signal values are attributed to 5′ end-missing early eluting impurities (Table S1). Moreover, the highest amount (relative to the main compound) was detected for impurities from 5′ N-18s to 5′ N-14s, as well as for 5′ N-18as and 5′ N-13as. These results suggest that coupling failures for the patisiran analogue are formed most significantly in the initial stages of synthesis.

#### 2.2.2. Antisense Strand of Patisiran

Changes of the elution order of impurities were observed for the CHOL stationary phase when two different pH values of the salt in the mobile phase were compared (Table 3), e.g., for 5′ N-16as, 3′ N-15as, 3′ N-8as, 3′ N-6as, 3′ N-7as. The majority of impurities, for which the elution order has changed (at pH 7.5 compared to 6.3), possess the additional phosphate group at the 3′ end (Table 3). This effect may suggest that it has a significant impact on the retention of OGNs when near-neutral mobile phase conditions are applied.

Regarding the retention data for CHOL obtained for pH 6.3, it should be pointed out that there is no strict linear relationship between the MW of impurities and their retention. However, certain trends can nevertheless be observed. For the antisense strand, OGNs with low molecular masses between 650–2000 Da (charge state −1 and −2) are generally the first to elute from the CHOL column (Table 3). This is followed by the elution of impurities with higher masses in the range of 2100–6300 Da (charge state −3 and −4) in the elution window between 16 to 19.5 min. The retention of OGNs in the reversed-phase system is the result of their total negative charge, size, and the change in polarity determined by the nucleobases. For this reason, for example, 5′ N-19as is eluted after 3′ N-18as and 5′ N-18as, which are more polar (Table 3).

Different tendencies were noticed when the ammonium formate of pH 7.5 was used in the mobile phase. First, eight impurities of small size (below 2000 Da) (charge state −1, −2) are eluted from the column. Then, only OGNs that have a phosphate group at the 3′ end (8 compounds) are eluted (between 12 and 14 min). Next, impurities that have an OH group at the 3′ end (10 compounds) are eluted from the CHOL column. Apparently, changing the pH of the mobile phase for the CHOL column actually alters the interactions and their strength between the stationary phase ligands and the OGNs; introducing an additional phosphate group into the OGN structure reduces its retention. This remains in line with theoretical knowledge. Increasing the pH reduces the positive charge on the stationary phase, resulting in reduced electrostatic interactions. Therefore, any additional ionized phosphate group will be stronger affected by this pH change and the cause of reduced OGN retention relative to impurities which miss this structural element.

These results clearly confirm that, in the case of mixed-mode stationary phases with hydrophilic-hydrophobic groups, the pH of the mobile phase is an additional parameter that can be changed to alter the separation selectivity by changing the retention of OGNs under RP conditions.

#### 2.2.3. Sense Strand of Patisiran

The retention of impurities for sense strand and pH 6.3 is based on the following order: first, the five OGNs with the lowest masses are eluted between 11 and 15 min, followed by compounds with masses from 2000–3000 Da (between 15 and 19 min), whereas the OGNs with the highest masses and charge states are eluted last (Table 3). These tendencies are quite analogous to those of the antisense strand and are based on similar effects.

For the sense strand, the impurities are mainly OGNs with an -OH group at the 3′ end. Only seven compounds have a 3′ phosphate terminal group (Table 3). Three of them are lower molecular weight compounds that (irrespective of the strand and the pH of the mobile phase) elute early, in a short time. Thus, for pH 7.5, there is no very clear dependence of retention on the type of terminal group at the 3′ end. Neither was there such a dependence of the elution order on the molecular weight or the oligo length.

### 2.3. Closely Related Impurities in the Main Peak of Sense and Antisense Strands

**Figure 3:**
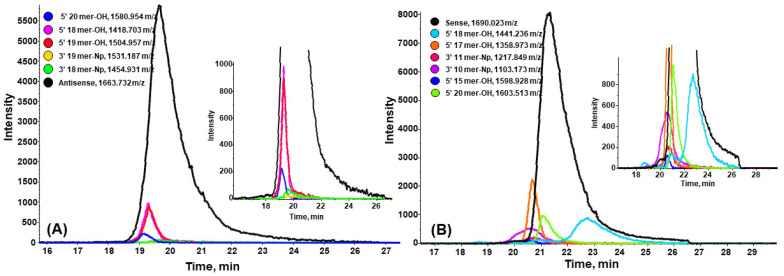
Extracted ion chromatograms (EIC) for selected ions eluted from CHOL column together with the main compound of: (**A**) antisense strand of patisiran analogue, (**B**) sense strand of patisiran analogue. Experimental LC conditions: MPA: 20 mM ammonium formate (pH 6.3); MPB: MeOH/20 mM ammonium formate (pH 6.3) 9:1 (*v*/*v*); 10–45 % MPB in 25 min (15 min re-equilibration); column temperature: 40 °C; autosampler temperature: 4 °C; flow rate: 0.3 mL/min; injection volume: 8 µL; MS parameters for nebulizer gas, heater gas, curtain gas, source temperature, ion spray voltage, declustering potential, and collision energy were set as follows: 90 psi, 90 psi, 35 psi, 550 °C, 4500 V, −150/−200/−150 V, −10 V.

The results summarized in Table 3 indicate that the complete separation of all impurities could not be achieved with CHOL. Some of them were eluted from the column together with the main compound (Table 3). Figure 3 presents the EIC for selected ions eluted from the CHOL column together with the antisense or sense strand. In the case of the antisense strand of patisiran, there are, in total, five N-1, N-2, N-3 shortmers differing in the 3′ end group (hydroxyl or phosphate) coeluting with the main compound: 5′ N-1as, 5′ N-2as, 5′ N-3as, 3′ N-2as, 3′ N-3as (Table 3). All of them are eluted in the front of the main peak (Figure 3A). Six impurities are eluted together with the main peak of patisiran sense strand, but one of them, 5′ N-3s, is eluted in the ‘tail’ of the main peak (Figure 3B). The other five impurities are the following shortmers: 3′ N-11s, 3′ N-10s, 5′ N-6s, 5′ N-4s, 5′ N-1s.

Although these structurally-related impurities were not able to be separated using the CHOL column, a characterization of those impurity species was still possible with a direct coupling with MS. To alleviate this selectivity problem, we decided to use 2D-LC in a selective comprehensive mode for this purpose to achieve a complete resolution.

### 2.4. Selective Comprehensive (High-Resolution Sampling) sRP × HILIC-2DLC for Patisiran

In our previous 2DLC experiments, reversed phase was employed as the second dimension for desalting before ESI-MS detection [22,23]. Hydrophilic interaction liquid chromatography (HILIC), which was shown to give enhanced separation under ion-pair free elution conditions, was envisaged as a second dimension for this 2DLC setup in combination with CHOL RP. We chose the previously developed HILIC method using a BEH Amide column for the present study [20]. The retention data are summarized in Table S2. Firstly, we have compared the normalized retention times (*t_N_*) obtained for HILIC and RP using CHOL to check the orthogonality of the systems, as 2D-LC performance depends on the separation orthogonality. The *t_N_* values were calculated by the use of the following formula:

(1) $$t_{N}=\frac{t_{R}-t_{0}}{t_{G}-t_{0}}$$

wherein *t_R_* is the retention time of the compound, *t_G_* the gradient time, and *t*_0_ is the void time.

**Figure 4:**
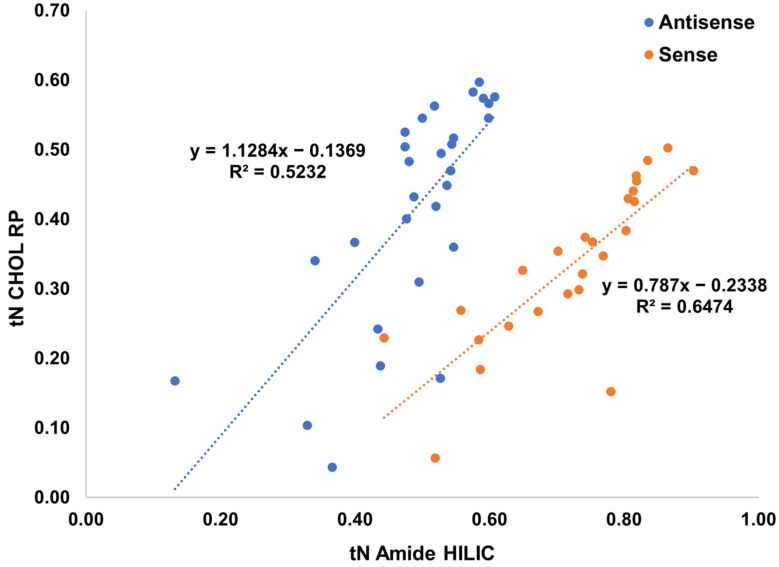
The correlation of normalized retention time (*t_N_*) between identical impurities and main compounds detected by HILIC with Amide column and RP with CHOL column for two strands separately.

The results on the orthogonality of the distinct LC modes are presented in Figure 4. The lower the determination coefficient (R^2^) value, the higher the degree of orthogonality between columns (values lower than 0.5 are considered as indicating a high degree of scatter, which is desirable for 2DLC). The R^2^ were equal to 0.5232 for the antisense strand and 0.6474 for the sense strand when the normalized retention time of corresponding impurities on the CHOL and BEH Amide columns were correlated (Figure 4). This indicates good orthogonality between both modes of LC and both columns, which is indispensable for a successful application of 2D-LC and can enable the selective separation of impurities in the second dimension.

**Figure 5:**
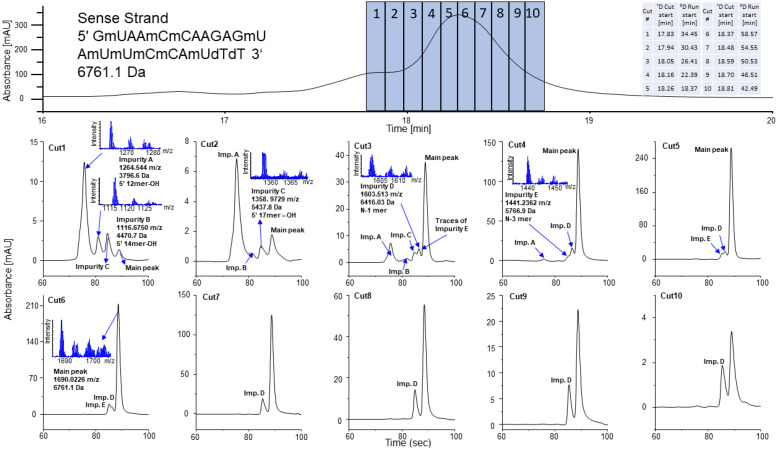
The UV chromatograms presenting the result of selective comprehensive 2D-LC analysis of sense strand of patisiran analogue. Upper chromatogram: ^1^D LC with the use of CHOL in RP mode; lower chromatograms: ^2^D LC with the use of Amide in HILIC mode. Detailed experimental conditions in Section 2.4.

Finally, the RP method with the use of CHOL column was used in the first dimension, whereas HILIC with BEH Amide was used in the second dimension. Several groups have reported about the selectivity of BEH Amide towards different kind of OGNs [19,20,35]. For this study, we chose basic conditions at pH 9 to enhance the ionization of the OGNs. With 15 mM ammonium acetate, both MS compatibility and good chromatographical performance are given. The selective comprehensive mode of 2D-LC was used in order to improve the separation of closely related impurities. In this mode, a certain part of the ^1^D chromatogram, herein covering the main peak, is comprehensively sampled by a number of adjacent fractions and transferred into the second dimension. In this work, 10 fractions were stored in 40 µL sampling loops until analysis by the ^2^D HILIC method. Due to the high aqueous content of the ^1^D RP eluent and its high elution strength in the ^2^D HILIC method, active solvent modulation was activated [36]. It allows dilution of the sampled fractions from ^1^D by weak ^2^D HILIC eluent before they are introduced onto the ^2^D HILIC column. It avoids peak distortions and sample breakthrough effects. The exemplary results for the sense strand of patisiran are presented in Figure 5 and Figure S11. Although it was not possible to obtain a full baseline separation of all six impurities eluted with the main peak in ^1^D by this sRP × HILIC-2DLC, the partial separation was sufficient to provide the required information on impurity determination in good quality. The use of HILIC in ^2^D allowed the separation of only three additional compounds (5′ N-4s, 5′ N-1s, 5′ N-3s) from the main sense strand (Figure 5). It is a limitation of the developed method that not all impurities could be fully baseline separated; yet, compared to a one-dimensional separation, an improved resolution could be achieved.

A change in the elution order was observed. The 5′ N-3s in HILIC was eluted before the sense strand and before the 5′ N-1s impurity, whereas in ^1^D, it was eluted after the main peak and other impurities. This effect is characteristic for these two chromatographic systems and further demonstrates the orthogonality of RP and HILIC.

Three of the impurities that were eluted together with the sense strand, 5′ N-6s (*m/z* 1598.9283), 3′ N-11s (*m/z* 1103.173), 3′ N-10s (*m/z* 1217.849), were not detected in the sRP × HILIC-2DLC approach due to their low concentration (100–500 cps in 1D-RPLC, see Figure 3B), splitting into multiple fractions and a further dilution effect upon injection into the ^2^D. This is the drawback of our method. The new multi-inject software solution from Agilent Technologies, which allows the transfer of the contents of the multiple loops of the multiple heartcutting loop decks at once before the entire deck is analyzed by a single ^2^D analysis cycle, could solve this problem, but was not yet available in this work [37]. It not only overcomes the sensitivity problem, but also saves time, since only one ^2^D analysis run needs to be carried out in contrast to the multiple injections of the different loops in the normal selective comprehensive 2DLC approach. This holds great promise for selective comprehensive sRP×HILIC-2DLC analysis of oligonucleotide pharmaceuticals, and will further empower the current selective comprehensive CHOL RP *×* BEH Amide HILIC-ESI-QTOF-MS approach in impurity profiling.

## 3. Materials and Methods

### 3.1. Materials

Acetic acid (ACS reagent, ≥99.8%), ammonium hydroxide (ACS reagent, 28–30%), ammonium acetate (LC-MS grade), and ammonium formate (LC-MS grade) were purchased from Sigma-Aldrich (Merck, Munich, Germany). LC-MS grade methanol (MeOH) and acetonitrile (ACN) were purchased from Carl Roth (Karlsruhe, Germany). Ultrapure water was obtained from Elga PurLab Ultra system (Celle, Germany). Both OGNs were purchased from Oligo Sigma (Merck, Munich, Germany) as desalted raw products without purification.

### 3.2. Oligonucleotides

Two OGNs were used during present study. Their sequences correspond to the sense and antisense strand of the patisiran, which is an active substance of a drug used for the treatment of polyneuropathy caused by hereditary transthyretin-mediated amyloidosis [38]. They were used as model compounds for the development of a method for the separation and identification of impurities. They were purchased as custom, synthesized, raw products, obtained as desalted, but non-purified, synthesis products from Oligo Sigma (Merck, Munich, Germany). Thus, they were not supposed to have drug quality, but were considered suitable test samples for our study. The sequence of passenger (sense) strand is as follows: 5′-G-Um-A-A-Cm-Cm-A-A-G-A-G-Um-A-Um-Um-Cm-Cm-A-Um-dT-dT-3′ with molecular mass of 6764 Da. The guide strand (antisense) has a sequence 5′-A-U-G-G-A-A-Um-A-C-U-C-U-U-G-G-U-Um-A-C-dT-dT-3′ and mass 6660 Da (dT stands for deoxyriboncleotide, whereas mU, mC stands for 2′-O-methylribonucleotide analogues).

### 3.3. Instrumentation

Preliminary, retention, and separation studies for cholesterol (CHOL) and alkylamide (AP) columns were performed with an Agilent 1290 Infinity II UHPLC system from Agilent Technologies (Waldbronn, Germany) equipped with a Multisampler (G7167B), binary High-Speed Pump (G7120A), column compartment (G7116B), and Diode Array Detector (DAD) (G7117B).

The 2D-LC experiments were carried out with the use of Agilent 1290 Infinity II 2D-LC Solution from Agilent Technologies, including a quaternary Flexible pump (G7104A), Multisampler (G7167B), and Variable Wavelength Detector (VWD) (G7114B) with 14 µL flow cell (G1314-60186) and a pressure relief valve (pressure release kit, G4236-600010) between the VWD and 2D-interface. The sampling frequency of the VWD was 5 Hz. In the second dimension, a binary High-Speed Pump (G7120A) and a Diode Array Detector (DAD) (G7117B) with 1 µL flow cell (G4212-60008) were employed. The sampling frequency of the DAD was 80 Hz. Two separate Infinity column compartments (G7116B) were used. Both dimensions were connected by a valve drive (G1170A) equipped with 5-position/10-port 2D-LC active solvent modulation (ASM) valve (#5067-4266) coupled to two 6-position/14-port valve heads (#5067-4142, multiple heart-cutting valves) equipped with six 40 µL loops each. The ASM valve contained the ASM capillary with dimensions of 85 × 0.12 mm (0.96 µL, #5500-1300) (ASM factor 5, ASM loop flushing: 3 times) [36]. The 2D-LC system was controlled by Agilent OpenLab CDS ChemStation Rev. C.01.10 with 2D-LC add-on software.

MS detection was performed on a SCIEX Triple TOF 5600+ QTOF mass spectrometer with a Duospray ion source operating in negative ESI mode. The 2D-LC and MS instruments were coupled with a contact closure connection for peripheral devices from SCIEX, and the MS instrument was controlled with Analyst TF 1.7 software (AB SCIEX, Darmstadt, Germany).

### 3.4. LC-MS Conditions

Three different columns have been used in the present study: the home-made CHOL and AP columns (125 × 2.1 mm, 5 µm particle size, 100 Å), as well as Acquity UPLC BEH Amide for the second dimension (50 × 2.1 mm, 1.7 µm particle size, 130 Å). The structures of stationary phases are presented in Figure 1. CHOL and AP were synthesized according to a previously described synthesis method [29,30]. They were prepared from the same batch of 5 μm Kromasil^®^ silica gel with 300 Å pore size. The CHOL stationary phase has 9.97% of carbon at the surface, whereas for AP, the carbon load is equal to 6.52%. The stationary phases were packed into stainless-steel columns with the use of a home-made apparatus with a Haskel pump (Burbank, CA, USA) under constant pressure (400 bars). Column void volume (*t*_0_) was measured by injecting methanol.

For the retention and separation studies with CHOL and AP columns, similar elution conditions were used. The chromatographic parameters were as follows: mobile phase A (MPA) was composed of salt solution, whereas mobile phase B (MPB) contained MeOH/salt solution (9:1; *v*/*v*); gradient elution consisted in 10–45% MPB change in 25 min (with 15 min re-equilibration); column temperature: 40 °C; flow rate: 0.3 mL/min; injection volume: 8 µL; spectrophotometric UV detection was performed at λ = 254 nm. Four different parameters were tested during this stage of studies, namely the pH of salt (4.5–7.5), salt concentration (10–40 mM), salt type (ammonium formate and acetate), and organic solvent type (MeOH, ACN).

Finally, the optimized conditions used in the ^1^D were as follows: column: CHOL; MPA: 20 mM ammonium formate (pH 6.3), MPB: MeOH/20 mM ammonium formate (pH 6.3) 9:1 (*v*/*v*); gradient elution: 10–45% MPB in 20 min (20 min re-equilibration); column temperature: 40 °C; autosampler temperature 10 °C; flow rate: 0.3 mL/min; injection volume: 8 µL.

The separation in the second dimension ^2^D was performed in a HILIC mode with the following final conditions: column—Acquity UPLC BEH Amide, MPA: 15 mM ammonium acetate in ACN/water 7:3 (*v*/*v*) (pH 9), MPB: 15 mM ammonium acetate (pH 9), gradient elution: hold 0% MPB for 0.33 min (ASM), 0–60% MPB in 2.38 min, re-equilibration for 1.69 min, column temperature: 60 °C, flow rate: 1.0 mL/min.

MS detection was carried out in a negative polarity mode with an ESI source. The MS parameters for RP experiments with CHOL were as follows: nebulizer gas, 90 psi; heater gas, 90 psi; curtain gas, 35 psi; source temperature, 550 °C; ion spray voltage, 4500 V; declustering potential, 200 V; and collision energy, 10 V. In the case of 2D experiments, these parameters were: nebulizer gas, 80 psi; heater gas, 80 psi; curtain gas, 40 psi; source temperature, 450 °C; ion spray voltage, 4500 V; declustering potential, 150 V; and collision energy, 10 V. The data processing was accomplished by using PeakView (AB Sciex, Darmstadt, Germany).

The identification of impurities was performed by assigning charge states of the ions observed at the full scan spectra, calculation of masses, or application of deconvolution. The PeakView equipped with Bio Tool Kit Plug-in (Version 2 2 0.11391, SCIEX) was used during the deconvolution (Figure S7). When the molecular mass of impurities was determined, their identification (sequence assignment) was performed by mass calculation with the use of RoboOligo and the OMA/OPA program [33,34]. Moreover, the structural annotation of impurities was also performed based on their fragmentation.

## 4. Conclusions

The results obtained during the present study confirmed the high potential of mixed hydrophobic/hydrophilic stationary phases for the study of OGN impurities. The RP HPLC mode was successfully used for the separation of impurities with a cholesterol (CHOL)-based stationary phase, and no ion-pair reagents were needed for that purpose. This is one of the advantages of the method developed during the study. Because both the alkylamide and cholesterol phases possess surface chemistry capable of changing properties depending on the pH of the mobile phase, this parameter proved to be crucial in developing a method for separating impurities from the parent compound. In both cases, the best separation results were obtained for pH in the range 6.5–7.5. The type and concentration of salt used in the mobile phase had a lower impact on the retention and separation compared to pH. However, it should be underlined that 20 mM ammonium formate gave the best results in terms of resolution. When comparing the impurity separation between the two stationary phases, a significantly higher efficiency was obtained for the cholesterol stationary phase (less peak broadening and higher retention compared to the alkylamide column). Each of these chromatographic parameters has an impact on the intensity of the OGN-derived signals at the mass spectra. However, the pH of the mobile phase was again the most influential, and the highest intensities of impurities ions were observed for pH equal 6.5.

For the final chromatographic method developed in RP HPLC using CHOL, a good separation of most impurities was achieved within 25 min. A few structurally closely related impurities were coeluted with the main sense or antisense strand. For the characterization of the majority of impurities, the developed CHOL 1DLC separation was sufficient. Our plans for the future are to reduce the diameter of CHOL stationary phase particles to 3 µm or less, which will certainly lead to an increase in the efficiency of impurities separation. It may then be possible to use only RP HPLC and control pH to alter retention and separation.

The good orthogonality between RP HPLC with CHOL and HILIC with a commercially available amide column allowed us to use 2D-LC in a selective comprehensive mode for the separation of closely related impurities. We achieved the separation of several impurities eluted together with the main OGN. Some minor impurities could not be detected anymore in the selective comprehensive 2DLC analysis due to splitting of the ^1^D peaks into several fractions and further dilution by ^2^D eluent. The multi-inject approach, which allows analysis of the entire loop deck at once before ^2^D analysis, has the potential to overcome this limitation and accelerate the entire analysis. With these further advancements, the developed 2D-LC method can be a successful tool for the separation and determination of impurities in oligonucleotide pharmaceuticals.

The main advantage of the developed method is the separation and identification of most impurities in 25 min using CHOL with RP HPLC. An advantage is the use of ion-pair-free or inorganic salts-free mobile phases. The use of 2D-LC leads to a further benefit: the increased resolution of compounds coeluted with the main OGN.

## Figures and Tables

**Table 1:** Exemplary results of retention factor (*k*) values determined for sense strand of patisiran analogue with regard to different chromatographic conditions applied during the study (under gradient elution: MPA: salt solution; MPB: MeOH/salt solution 9:1 (*v*/*v*); gradient elution: 10–45% MPB in 20 min).

Stationary Phase	*k*			
pH of 20 mM ammonium formate				
	4.5	5.5	6.3	7.5
CHOL	-	16.62 ± 0.21	15.23 ± 0.16	13.09 ± 0.09
AP	-	13.22 ± 0.30	9.93 ± 0.02	7.15 ± 0.02
Concentration of ammonium formate				
	10 mM	20 mM	40 mM	
CHOL	10.44 ± 0.11	15.23 ± 0.27	13.96 ± 0.20	
AP	8.08 ± 0.18	9.93 ± 0.13	9.12 ± 0.08	
Type of salt				
	20 mM ammonium formate		20 mM ammonium acetate	
CHOL	15.23 ± 0.14		10.98 ± 0.25	
AP	9.93 ± 0.09		7.72 ± 0.13	
Type of organic solvent				
	MeOH		ACN	
CHOL	15.23 ± 0.29		14.17 ± 0.22	
AP	9.93 ± 0.018		8.18 ± 0.21	

**Table 2:** Peak areas for selected ions of extracted ion chromatograms (EIC).

Ion (*m/z*)	EIC Peak Area	
	pH 6.3	pH 7.5
	Sense strand	
865.173	236,618 ± 1302	81,850 ± 591
1235.711	151,654 ± 973	68,514 ± 462
1155.188	66,958 ± 664	51,524 ± 349
1489.9089	87,257 ± 471	32,500 ± 588
1690.023	501,090 ± 1364	259,873 ± 1147
	Antisense strand	
	pH 6.3	pH 7.5
850.172	198,963 ± 1197	71,599 ± 759
902.147	201,337 ± 1372	98,004 ± 1024
1074.673	73,951± 1005	33,820 ± 1153
1247.197	89,620 ± 826	38,391 ± 944
1663.732	296,281 ± 1277	139,009 ± 1039

**Table 3:** Impurities identified in antisense and sense patisiran strands during their RP HPLC analysis with the use of CHOL stationary phase.

*m/z* Values for the Most Abundant Ion	Charge	Impurity	Impurity Sequence	Deconvoluted Mass (Da)	Retention Time CHOL pH 6.3 (min)	Retention Time CHOL pH 7.5 (min)
ANTISENSE STRAND						
652.082	−1	3′ N-19as	5′ AU 3′—PO	653.082	8.46	5.06
979.118	−1	3′ N-18as	5′ AUG 3′—cyc	980.118	10.76	8.68
850.170	−1	5′ N-18as	5′ CdTdT 3′—OH	851.170	10.73	9.06
545.129	−1	5′ N-19as	5′ dTdT 3′—OH	546.129	11.15	9.94
749.128	−2	5′ N-16as	5′ mUACdTdT 3′—OH	1500.257	13.95	11.64
835.110	−2	3′ N-16as	5′ AUGGA 3′—PO	1672.221	14.03	9.33
902.141	−2	5′ N-15as	5′ UmUACdTdT 3′—OH	1806.282	14.06	11.56
1074.665	−2	5′ N-14as	5′ GCmUACdTdT 3′—OH	2151.330	15.62	13.01
999.632	−2	3′ N-15as	5′ AUGGAA 3′—PO	2001.264	16.11	10.73
1035.139	−3	5′ N-11as	5′ UUGGUmUACdTdT 3′—OH	3108.418	16.35	13.6
831.122	−3	5′ N-13as	5′ GGUmUACdTdT 3′—OH	2496.367	16.8	14.23
1136.820	−3	5′ N-10as	5′ CUUGGUmUACdTdT 3′—OH	3413.459	17.04	14
1043.630	−4	3′ N-8as	5′ AUGGAAmUACUCUU 3′—PO	4178.519	17.28	12.52
967.3734	−4	3′ N-9as	5′ AUGGAAmUAUUUU 3′—PO	3873.496	17.28	12.51
928.867	−4	5′ N-9as	5′ UCUUGGUmUACdTdT 3′-OH	3719.470	17.46	14.12
1005.132	−4	5′ N-8as	5′ CUCUUGGUmUACdTdT 3′—OH	4024.528	17.46	13.78
882.455	−3	3′ N-13as	5′ AUGGAAmUA 3′—PO	2650.364	17.66	12.56
1187.822	−3	3′ N-10as	5′ AUGGAAmUACUC 3′—PO	3566.466	17.94	12.66
1167.402	−4	5′ N-6as	5′ mUACUCUUGGUmUACdTdT 3′—OH	4673.609	18.21	14.16
984.135	−3	3′ N-12as	5′ AUGGAAmUAC 3′—PO	2955.406	18.41	12.83
1129.892	−4	3′ N-7as	5′ AUGGAAmUACUCUUG 3′—PO	4523.570	18.7	13.13
1249.665	−4	5′ N-5as	5′ AmUACUCUUGGCmUACdTdT 3′—OH	5002.661	18.95	14.23
1216.152	−4	3′ N-6as	5′ AUGGAAmUACUCUUGG 3′—PO	4868.606	19.04	13.55
1580.954	−4	5′ N-1as	5′ UGGAAmUACUCUUGGUmUACdTdT 3′—OH	6327.814	19.16	15.28
1418.703	−4	5′ N-3as	5′ GAAmUACUCUUGGUmUACdTdT 3′—OH	5676.783	19.29	15.46
1504.957	−4	5′ N-2as	5′ GGAAmUACUCUUGGUmUACdTdT 3′—OH	6021.828	19.33	15.47
1531.187	−4	3′ N-2as	5′ AUGGAAmUACUCUUGGUmUAC 3′—PO	6128.748	19.54	15.14
1454.931	−4	3′ N-3as	5′ AUGGAAmUACUCUUGGUmUA 3′—PO	5823.723	19.74	14.92
**1663.732**	**−4**	**Antisense**	**5′ AUGGAAmUACUCUUGGUmUACdTdT 3′—OH**	**6656.866**	**20.03**	**15.68**
**SENSE STRAND**						
1011.145	−1	3′ N-18s	5′ GmUA 3′—PO	1012.130	11.77	7.49
865.169	−1	5′ N-18s	5′ mUdTdT 3′—OH	866.169	13.58	11.94
1340.212	−1	3′ N-17s	5′GmUAA 3′—PO	1341.212	14.49	9.42
915.667	−2	5′ N-15s	5′ mCmCAmUdTdT 3′—OH	1833.337	15.12	12.44
756.134	−2	5′ N-16s	5′ mCAmUdTdT 3′—OH	1514.268	15.18	12.94
1075.689	−2	5′ N-14 OHs	5′ mUmCmCAmUdTdT 3′—OH	2153.353	16.18	13.22
1115.676	−2	5′ N-14 POs	5′ mUmCmCAmUdTdT 3′—PO	2233.352	16.67	16.62
1235.714	−2	5′ N-13s	5′ mUmUmCmCAmUdTdT 3′—OH	2473.396	17.21	14.12
1153.181	−2	3′ N-14s	5′ GmUAAmCmCA 3′—PO	2308.362	17.92	15.7
1395.741	−2	5′ N-12 (A-mU)s	5′ mUmUmUmCmCAmUdTdT 3′—OH	2793.442	18.26	14.9
933.150	−3	5′ N-12s	5′ AmUmUmCmCAmUdTdT 3′—OH	2802.450	18.65	15.33
1039.831	−3	5′ N-11s	5′ mUAmUmUmCmCAmUdTdT 3′—OH	3122.492	18.79	15.48
1317.703	−2	3′ N-13s	5′ GmUAAmCmCAA 3′—Np	2637.407	18.87	12.17
1154.857	−3	5′ N-10s	5′ GmUAmUmUmCmCAmUdTdT 3′—OH	3467.530	19.14	15.7
1264.544	−3	5′ N-9s	5′ AGmUAmUmUmCmCAmUdTdT 3′—OH	3796.590	20.31	16.95
1489.237	−3	5′ N-7s	5′ AGAGmUAmUmUmCmCAmUdTdT 3′—OH	4470.688	20.4	16.63
1598.928	−3	5′ N-6s	5′ AAGAGmUAmUmUmCmCAmUdTdT 3′—OH	4799.718	20.58	16.96
1103.173	−3	3′ N-11s	5′ GmUAAmCmCAAGA 3′—PO	3311.495	20.62	13.39
1217.849	−3	3′ N-10s	5′ GmUAAmCmCAAGAG 3′—PO	3656.547	20.69	13.59
1358.973	−4	5′ N-4s	5′ mCmCAAGAGmUAmUmUmCmCAmUdTdT 3′—OH	5437.841	20.68	16.58
1603.513	−4	5′ N-1s	5′ mUAAmCmCAAGAGmUAmUmUmCmCAmUdTdT 3′—OH	6416.030	21.08	17.07
**1690.023**	**−5**	**Sense (guide strand)**	**5′ GmUAAmCmCAAGAGmUAmUmUmCmCAmUdTdT 3′—OH**	**6761.087**	**21.8**	**17.17**
1441.236	−4	5′ N-3s	5′ AmCmCAAGAGmUAmUmUmCmCAmUdTdT 3′—OH	5766.896	22.71	17.03

## Data Availability

Not applicable.

## Supplementary Materials

The following supporting information can be downloaded at: https://www.mdpi.com/article/10.3390/ijms232314960/s1.
