# Supplementary material for: Cholesterol Stationary Phase in the Separation and Identification of siRNA Impurities by Two-Dimensional Liquid Chromatography-Mass Spectrometry
Source: Int J Mol Sci. 2022 Nov 29;23(23):14960. doi: 10.3390/ijms232314960 (PMC9738757; doi:10.3390/ijms232314960)
Supplement: Supplementary file 1 [file ijms-23-14960-s001.zip › ijms-2029830-supplementary.pdf]

Cholesterol stationary phase in the separation and identification of siRNA impurities by two-dimensional liquid chromatography-mass spectrometry

Sylwia Studzińska [1,2,*], Feiyang Li [2], Michał Szumski [1], Michael Lämmerhofer [2], Bogusław Buszewski [1]

[1] Chair of Environmental Chemistry and Bioanalytics, Faculty of Chemistry, Nicolaus Copernicus University in Toruń, 7 Gagarin Str., PL-87-100 Toruń, Poland

[2] Institute of Pharmaceutical Sciences, Pharmaceutical (Bio-)Analysis, University of Tübingen, Auf der Morgenstelle 8, 72076 Tübingen, Germany

[3] Centre for Modern Interdisciplinary Technologies, Nicolaus Copernicus University in Toruń, 4 Wilenska St., 87-100 Toruń, Poland

***** Correspondence: kowalska@chem.umk.pl; Tel.: +48 56 6114753

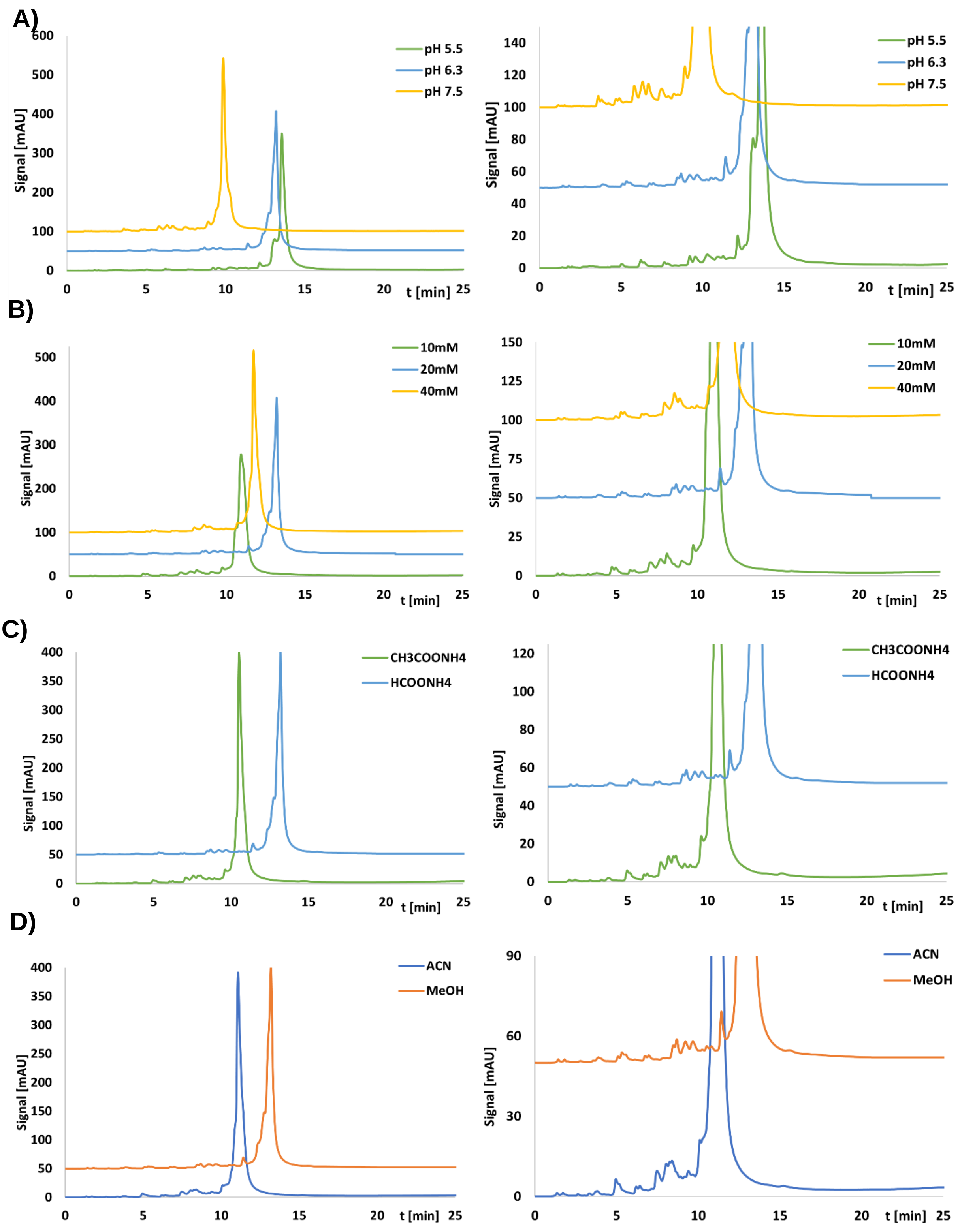

Figure S1. Exemplary chromatograms (left side) with enlarged view (right side) for sense strand of patisiran analogue for AP stationary phase obtained for: A) different pH values (for 20mM ammonium formate), B) different salt concentration (for ammonium formate); C) two different salts (both of them were 20mM solutions); D) two different organic solvents. Experimental conditions: MPA: salt solution, MPB: MeOH/salt solution 9:1 (v/v); gradient elution: 10-45% MPB in 20 minutes (20 minutes re-equilibration); column temperature: 40 °C; autosampler temperature 10 °C; flow rate: 0.3 mL/min; injection volume 8µL.

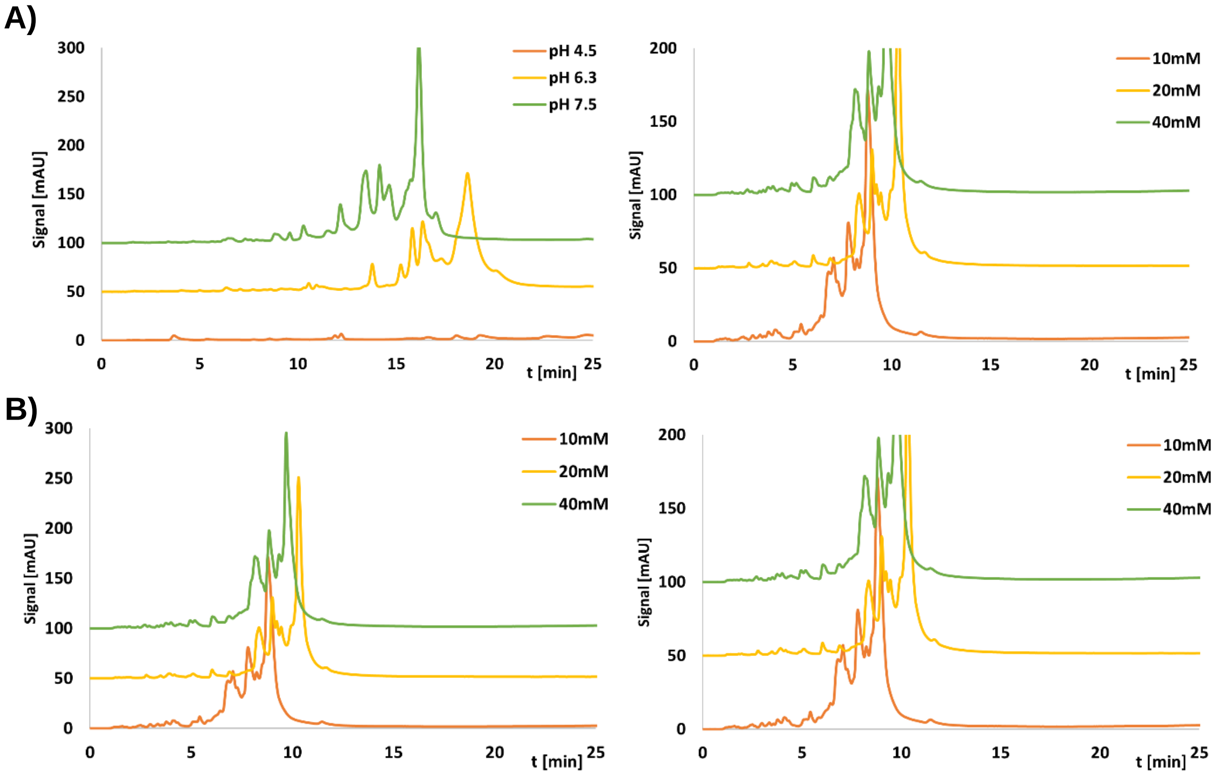

Figure S2. Exemplary chromatograms (left side) with enlarged view (right side) for antisense strand of patisiran analogue for: A) CHOL, different pH values (for 20mM ammonium formate), B) AP, different salt concentration (for ammonium formate, pH 6.3). Experimental conditions: MPA: ammonium formate, MPB: MeOH/20 mM ammonium formate 9:1 (v/v); gradient elution: 10-45% MPB in 20 minutes (20 minutes re-equilibration); column temperature: 40 °C; autosampler temperature 10 °C; flow rate: 0.3 mL/min; injection volume 8µL.

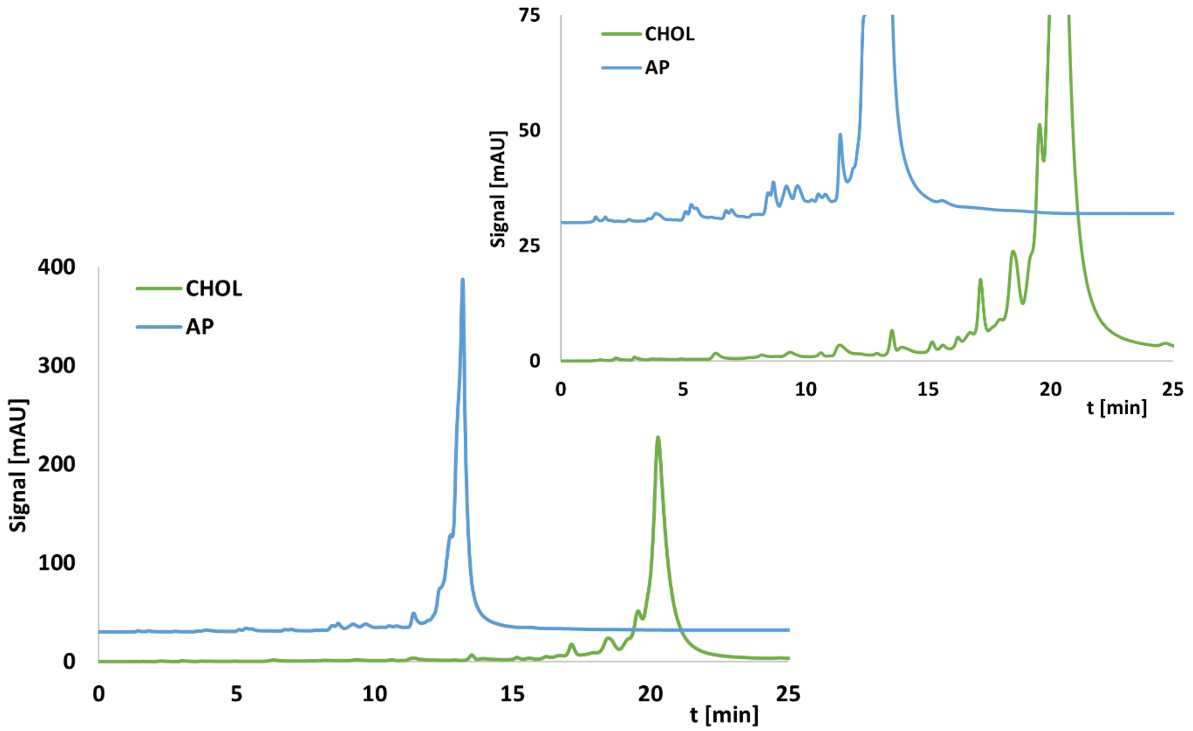

Figure S3. Chromatograms of the separation of the sense strand oligonucleotide and its impurities for two different columns and optimized conditions. Experimental conditions: MPA: 20 mM ammonium formate (pH 6.3), MPB: MeOH/20 mM ammonium formate (pH 6.3) 9:1 (v/v); gradient elution: 20 mM ammonium formate (pH 6.3); column temperature: 40 °C; autosampler temperature 10 °C; flow rate: 0.3 mL/min; injection volume 8 μL; UV detection with λ=254nm.

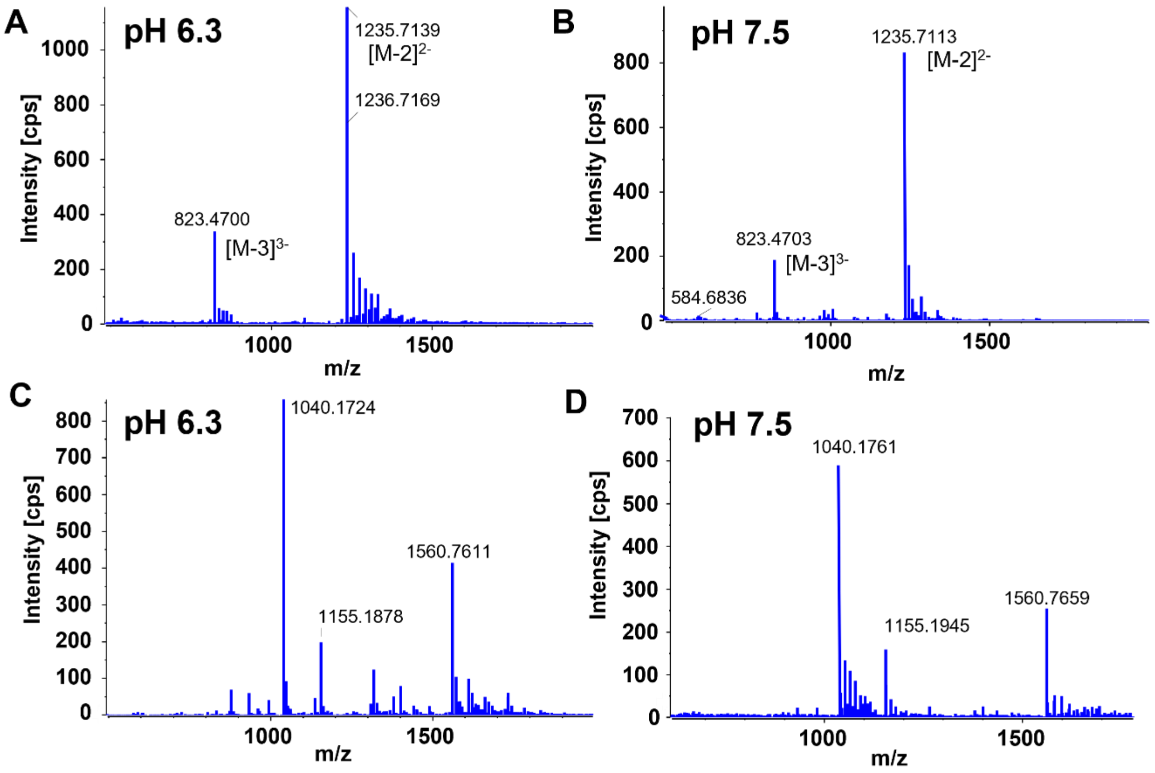

Figure S4. The full scan MS spectra for two impurities 5' N-13 (A, B) and 5' N-11 (C, D) and mobile phases of pH = 6.3 (A, C) and 7.5 (B, D). Experimental conditions: MPA: 20 mM ammonium formate (pH 6.3), MPB: MeOH/20 mM ammonium formate (pH 6.3) 9:1 (v/v); gradient elution: 10-45% MPB in 20 minutes (20 minutes re-equilibration); column temperature: 40°C; autosampler temperature 10°C; flow rate: 0.3 mL/min; injection volume 8μL; Q-TOF-MS conditions: nebulizer gas 90 psi, heater gas 90 psi, curtain gas 35 psi, source temperature 550 °C, ion spray voltage 4500 V, declustering potential -200 V and collision energy -10 V.

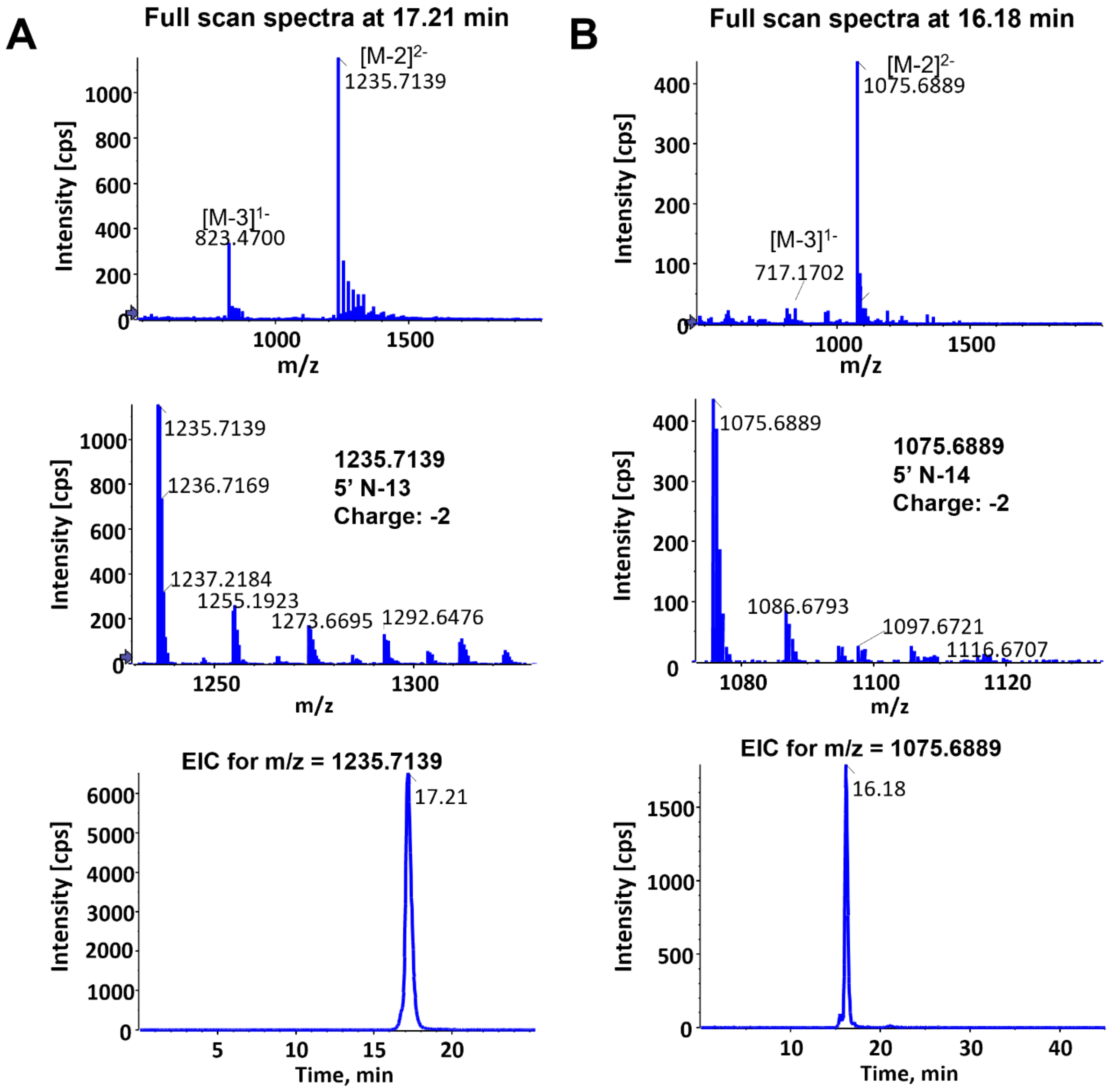

Figure S5. RP-LC-MS spectra and EIC of two selected impurity species from sense strand of Patisiran analogue: A) 5' N-13 at 17.21 min., B) 5' N-14 at 16.18 min. Experimental conditions: column: CHOL; MPA: 20 mM ammonium formate (pH 6.3), MPB: MeOH/20 mM ammonium formate (pH 6.3) 9:1 (v/v); gradient elution: 10-45% MPB in 20 minutes (20 minutes re-equilibration); column temperature: 40 °C; autosampler temperature 10 °C; flow rate: 0.3 mL/min; injection volume 8µL; MS parameters: nebulizer gas 90 psi, heater gas 90 psi, curtain gas 35 psi, source temperature 550 °C, ion spray voltage 4500 V, declustering potential -200 V and collision energy -10 V.

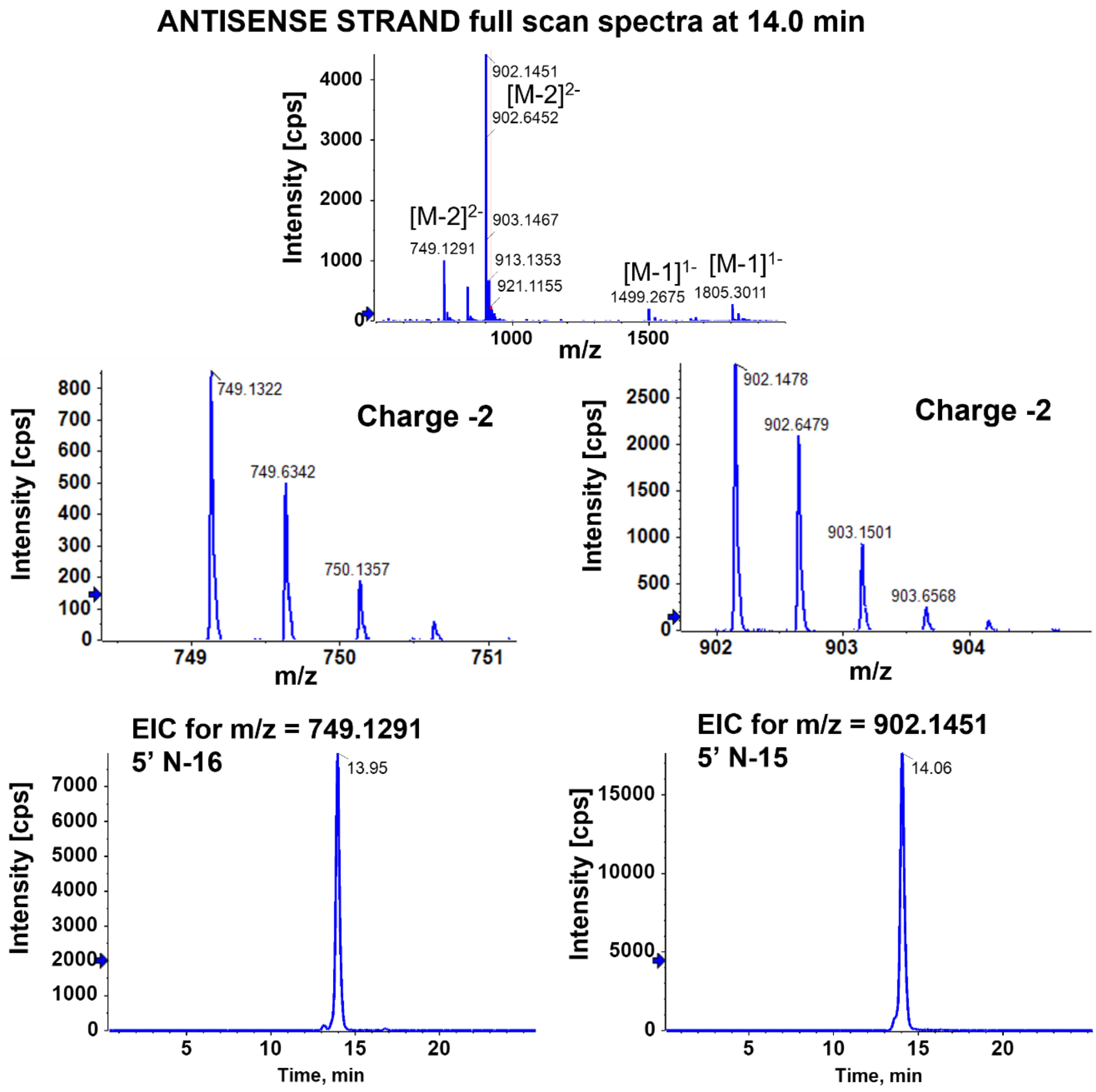

Figure S6. RP-LC-MS spectra and EIC of two selected impurity species from antisense strand of patisiran analogue. Experimental conditions: column: CHOL; MPA: 20 mM ammonium formate (pH 6.3), MPB: MeOH/20 mM ammonium formate (pH 6.3) 9:1 (v/v); gradient elution: 10-45% MPB in 20 minutes (20 minutes re-equilibration); column temperature: 40 °C; autosampler temperature 10 °C; flow rate: 0.3 mL/min; injection volume 8μL; MS parameters: nebulizer gas 90 psi, heater gas 90 psi, curtain gas 35 psi, source temperature 550 °C, ion spray voltage 4500 V, declustering potential -200 V and collision energy -10 V.

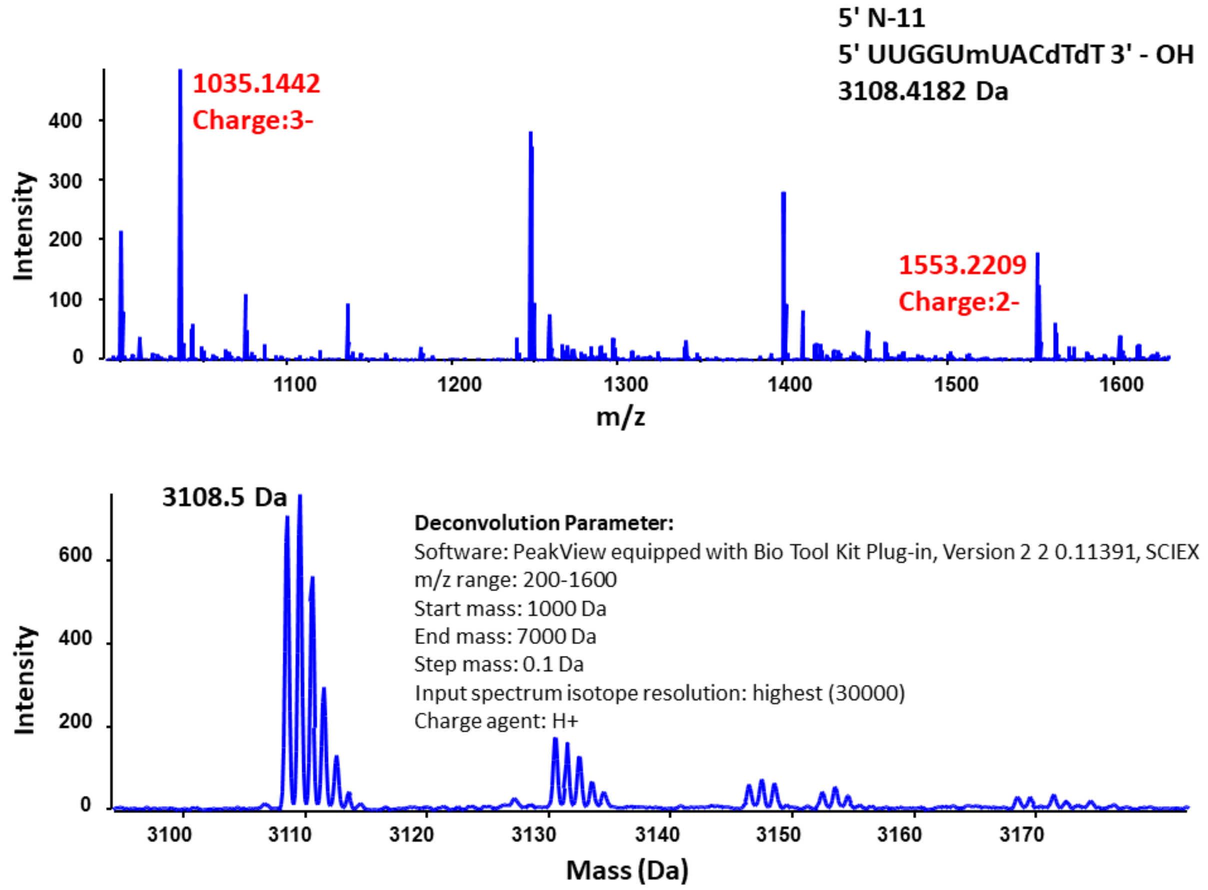

Figure S7. The scheme of deconvolution procedure used during impurities identification.

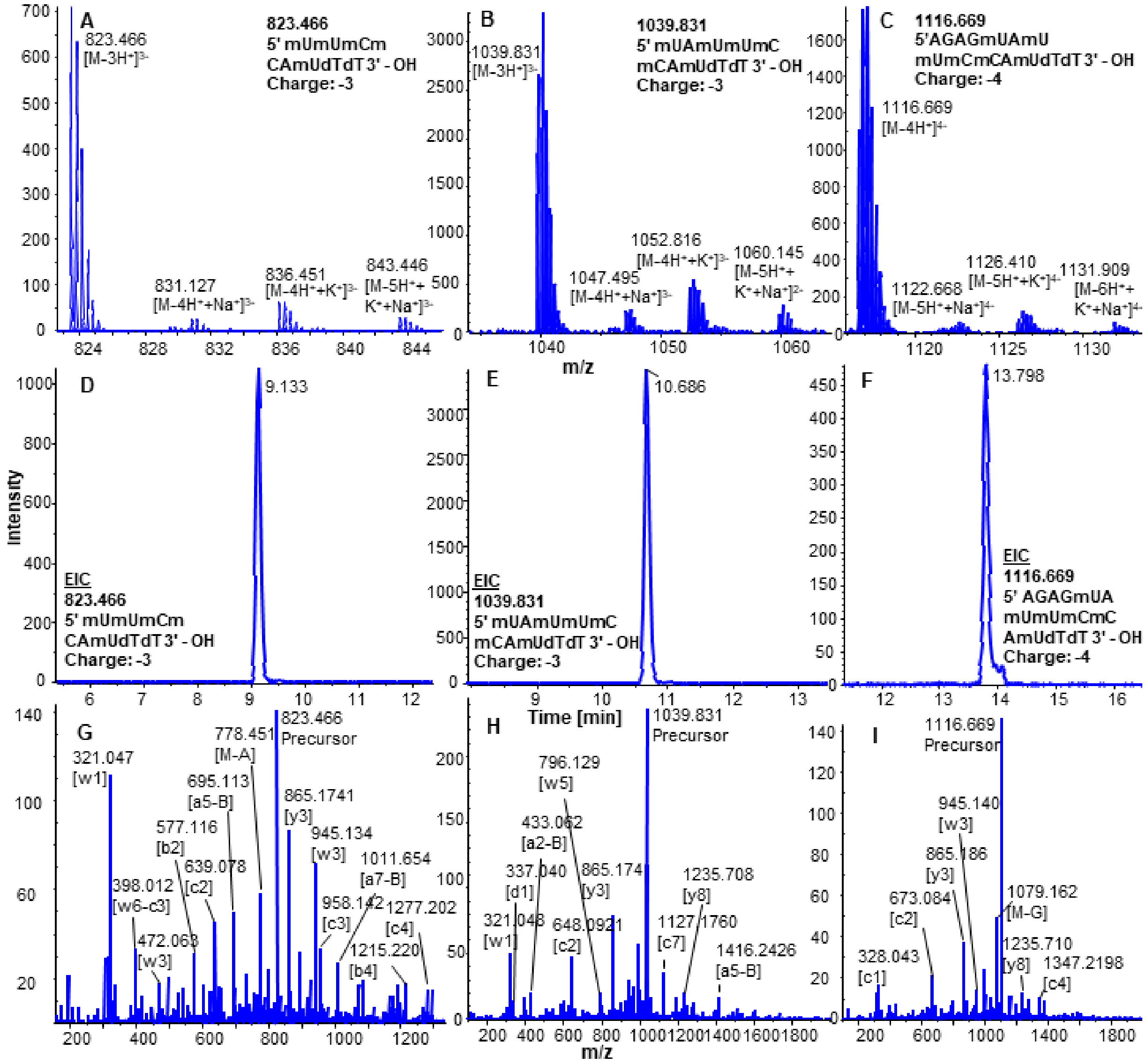

Figure S8. HILIC-LC-MS/MS spectra and extracted ion chromatogram of selected impurity species from sense strand of patisiran analogue. (A-C): MS spectra of impurity species with the most abundant charge states; (D-F): Extracted ion chromatograms of the monoisotopic mass; (G-I): MS^2 spectra of the corresponding precursor ion.

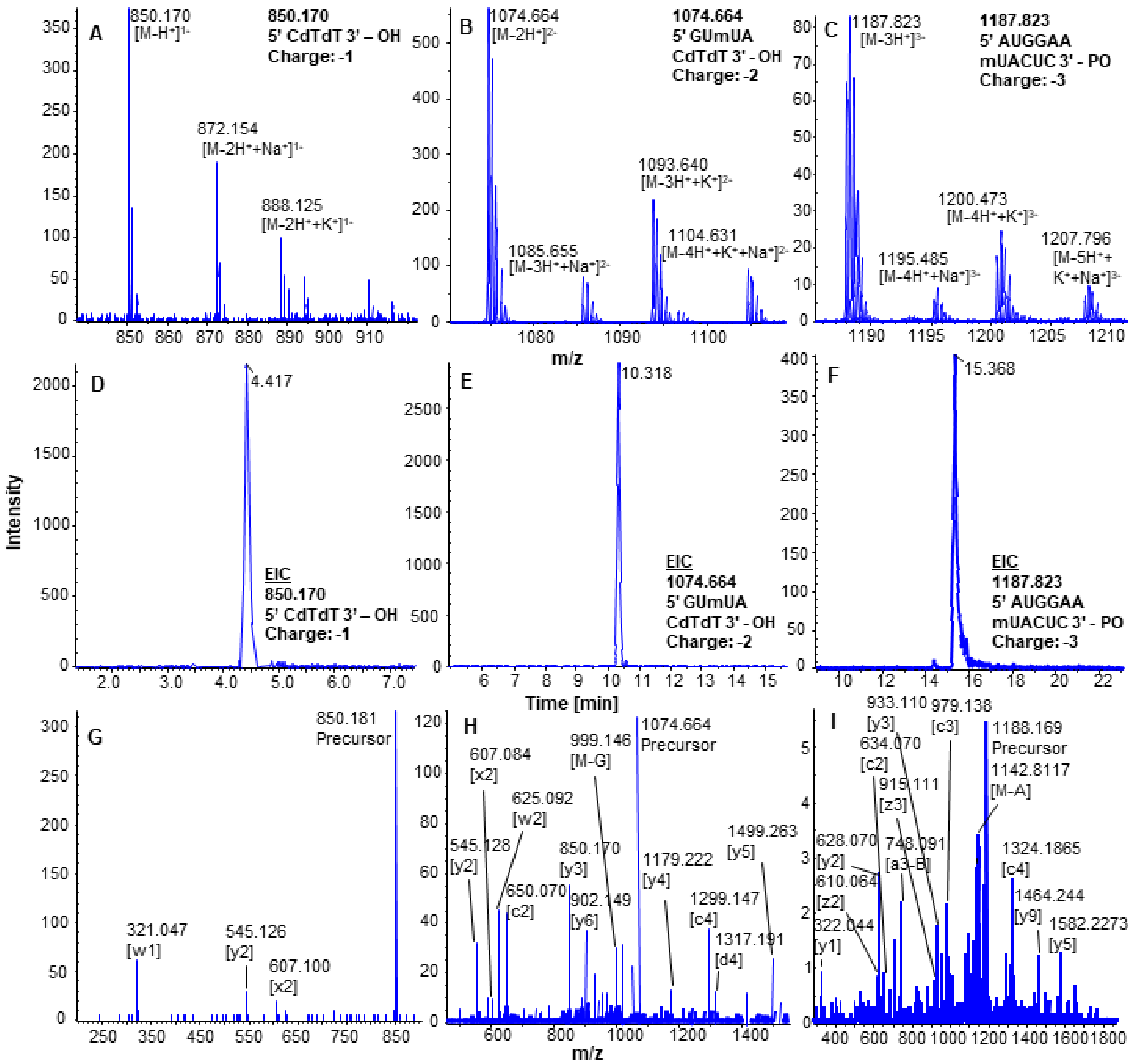

Figure S9. HILIC-LC-MS/MS spectra and extracted ion chromatogram of selected impurity species from antisense strand of patisiran analogue. (A-C): MS spectra of impurity species with the most abundant charge states; (D-F): Extracted ion chromatograms of the monoisotopic mass; (G-I): MS^2 spectra of the corresponding precursor ion.

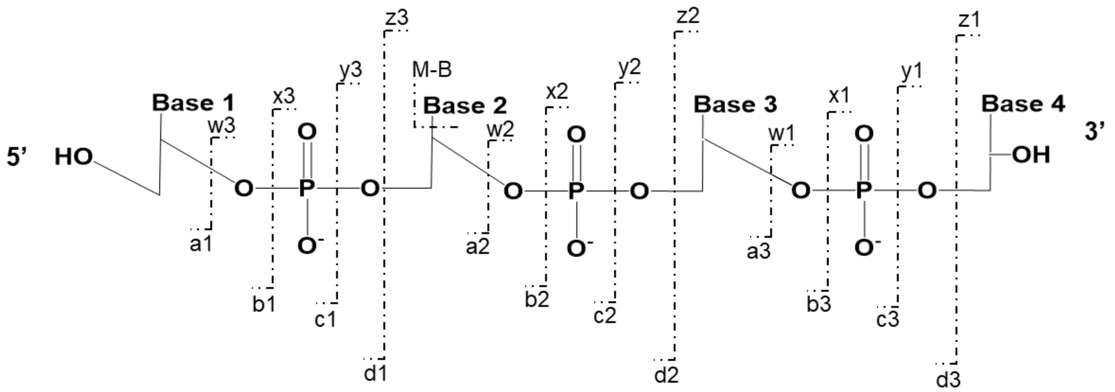

Figure S10. The standard nomenclature for oligonucleotide fragmentation during in mass spectrometry. The numbering of a-B, b, c, d and w, y fragments starts from the 5’ end and 3’ end.

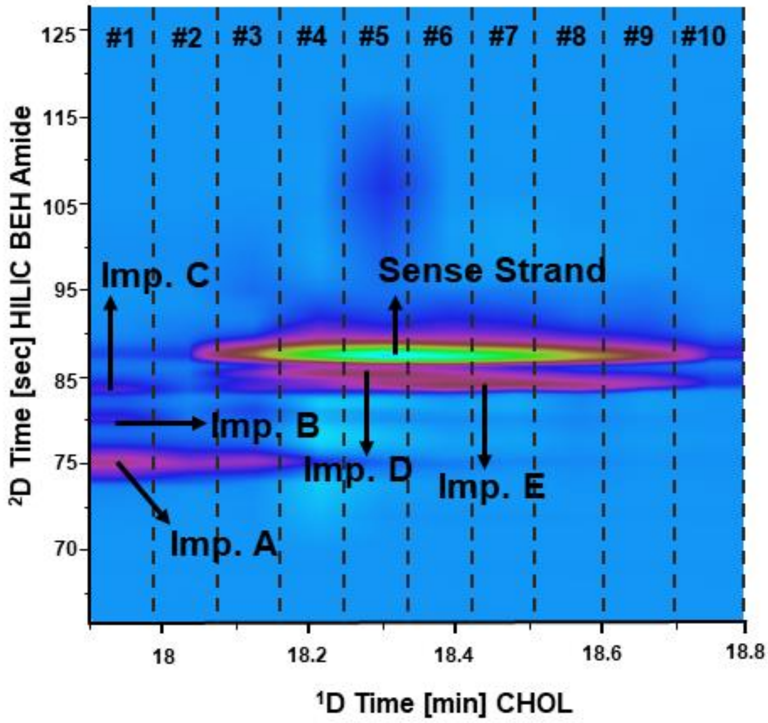

Figure S11. Heat map of the high-resolution sampling 2D-LC of the sense strand with ESI-MS detection.

Table S1. The relative proportion for each impurity sequence determined as peak area and relative peak area for both MS and UV detection and CHOL column (with mobile phase of pH 6.3).

The *m/z* values for most abundant ion	Charge	Impurity	Deconvoluted mass [Da]	Retention time CHOL pH 6.3 [min]	Peak number	MS detection		UV detection	
						Peak area	Relative to main [%]	Peak Area	Relative to main [%]
**ANTISENSE STRAND**									
652.082	-1	3' N-19as	653.082	8.46	1	12183	8.30	26.8	4.38
979.118	-1	3' N-18as	980.118	8.68	2	1245	0.85	20.5	3.35
850.170	-1	5' N-18as	851.170	10.73	3	5.60E+04	38.19	33.2	5.42
545.130	-1	5' N-19as	546.129	11.15	4	6.54E+04	44.61	32.4	5.29
749.128	-2	5' N-16as	1500.257	13.95	5	44526	30.35	494.3	80.77
835.111	-2	3' N-16as	1672.221	14.03	6	49860	33.99	co-eluted with 5	-
902.141	-2	5' N-15as	1806.282	14.06	7	105901	72.19	co-eluted with 5	-
1074.665	-2	5' N-14as	2151.330	15.62	8	72189	49.21	515.9	84.30
999.632	-2	3' N-15as	2001.264	16.11	9	22658	15.45	2502.2	408.86
1035.139	-3	5' N-11as	3108.418	16.35	10	39532	26.95	co-eluted with 9	-
831.123	-3	5' N-13as	2496.368	16.8	11	18017	12.28	co-eluted with 9	-
1136.819	-3	5' N-10as	3413.459	17.04	12	22041	15.02	co-eluted with 9	-
967.374	-4	3' N-9as	3873.496	17.28	13	1089	0.74	co-eluted with 9	-
1043.630	-4	3' N-8as	4178.519	17.28	14	1191	0.81	co-eluted with 9	-
928.868	-4	5' N-9as	3719.470	17.46	15	358	0.24	co-eluted with 9	-
1005.132	-4	5' N-8as	4024.528	17.46	16	1020	0.70	co-eluted with 9	-
882.455	-3	3' N-13as	2650.364	17.66	17	5200	3.54	co-eluted with 9	-
1187.822	-3	3' N-10as	3566.466	17.94	18	10870	7.41	co-eluted with 9	-
1167.402	-4	5' N-6as	4673.609	18.21	19	2881	1.96	co-eluted with 9	-
984.136	-3	3' N-12as	2955.407	18.41	20	6696	4.56	1005.1	164.23
1129.893	-4	3' N-7as	4523.570	18.7	21	1804	1.23	co-eluted with 20	-
1249.665	-4	5' N-5as	5002.661	18.95	22	5223	3.56	co-eluted with 20	-
1216.152	-4	3' N-6as	4868.606	19.04	23	3009	2.05	co-eluted with 20	-

1580.954	-4	5' N-1as	6327.814	19.16	24	1965	1.34	co-eluted with 20	-
1418.703	-4	5' N-3as	5676.783	19.29	25	8383	5.71	co-eluted with 20	-
1504.96	-4	5' N-2as	6021.828	19.33	26	9110	6.21	co-eluted with main	-
1531.187	-4	3' N-2as	6128.748	19.54	27	417	0.28	co-eluted with main	-
1454.931	-4	3' N-3as	5823.723	19.74	28	612	0.42	co-eluted with main	-
1663.732	-4	**Antisense strand**	6656.866	20.03	29	146700	100.00	7503.4	1226.05
**SENSE STRAND**									
1011.145	-1	3' N-18s	1012.123	11.77	1	24971	11.67	85.4	6944.67
865.169	-1	5' N-18s	866.169	13.58	2	72256	33.77	166.7	13555.93
1340.212	-1	3' N-17s	1341.212	14.49	3	5581	2.61	29.3	2382.66
915.667	-2	5' N-15s	1833.337	15.12	4	9146	4.27	co-eluted with 3	-
756.134	-2	5' N-16s	1514.268	15.18	5	7261	3.39	co-eluted with 3	-
1075.689	-2	5' N-14 OHs	2153.353	16.18	6	13770	6.44	102.4	8327.10
1115.676	-2	5' N-14 POs	2233.352	16.67	7	1778	0.83	117.4	9546.88
1235.714	-2	5' N-13s	2473.396	17.21	8	57222	26.74	89.8	7302.47
1153.181	-2	3' N-14s	2308.362	17.92	9	22286	10.42	74.9	6090.81
1395.750	-2	5' N-12 (A-mU)	2793.442	18.26	10	1459	0.68	co-eluted with 9	-
933.14	-3	5' N-12s	2802.449	18.65	11	2346	1.10	103.8	8440.94
1039.831	-3	5' N-11s	3122.492	18.79	12	22268	10.41	co-eluted with 11	-
1317.703	-2	3' N-13s	2637.407	18.87	13	10246	4.79	co-eluted with 11	-
1154.857	-3	5' N-10s	3467.530	19.14	14	27656	12.93	137.6	11189.53
1264.544	-3	5' N-9s	3796.590	20.31	15	32423	15.15	357.7	29087.91
1489.237	-3	5' N-7s	4470.688	20.4	16	18238	8.52	261.9	21297.52
1598.928	-3	5' N-6s	4799.718	20.58	17	988	0.46	794.3	64591.91
1103.173	-3	3' N-11s	3311.495	20.62	18	12452	5.82	co-eluted with 17	-
1358.973	-4	5' N-4s	5437.841	20.68	19	18992	8.88	co-eluted with 17	-
1217.849	-3	3' N-10s	3656.547	20.69	20	3450	1.61	co-eluted with 17	-
1603.513	-4	5' N-1s	6416.030	21.08	21	12325	5.76	co-eluted with 17	-
1690.023	-5	**Sense strand**	6761.087	21.8	22	213956	100.00	10316.5	838930.46
1441.236	-4	5' N-3s	5766.896	22.71	23	24133	11.28	1498.4	121848.82

Table S2. The impurities identified with the use of HILIC for sense and antisense strands of patisiran analogue together with the retention times used for orthogonality studies.

**ANTISENSE**				**SENSE**			
m/z	**Charge state of most abundant ion**	**Time [min]**	**Name**	***m/z***	**Charge state of most abundant ion**	**Time [min]**	**Name**
545.129	-1	2.696	5' N-19as	865.169	-1	3.082	5' N-18s
850.170	-1	4.424	5' N-18as	865.169	-1	3.31	5' N-18s
652.082	-1	6.266	3' N-19as	865.169	-1	3.704	5' N-18s
979.118	-1	6.37	3' N-18as	756.134	-2	6.73	5' N-16s
749.128	-2	6.888	5' N-16as	915.669	-2	7.969	5' N-15s
902.141	-2	8.41	5' N-15as	505.065	-2	8.045	3' N-18s
1074.665	-2	10.342	5' N-14as	716.784	-3	8.522	5' N-14s
835.110	-2	11.237	3' N-16as	823.466	-3	9.131	5' N-13s
831.126	-3	11.8	5' N-13as	669.593	-2	9.185	3' N-17s
999.632	-2	11.986	3' N-15as	930.147	-3	9.868	5' N-12s (mU-A)
882.455	-3	12.961	3' N-13as	933.150	-3	10.037	5' N-12s
1035.139	-3	13.481	5' N-11as	1039.831	-3	10.684	5' N-11s
984.136	-3	13.867	3' N-12as	1115.666	-2	10.839	5' N-14 POs
1136.820	-3	14.349	5' N-10as	1153.174	-2	11.621	3' N-14s
928.868	-4	14.958	5' N-9as	865.882	-4	12.005	5' N-10s
1187.822	-3	15.334	3' N-10as	878.131	-3	12.201	3' N-13s
1005.132	-4	15.66	5' N-8as	1264.53	-3	12.47	5' N-9s
967.374	-4	15.937	3' N-9as	1264.53	-3	12.8	5' N-9 (isomer)s
1167.403	-4	16.038	5' N-6as	1102.832	-3	13.669	3' N-11s
1249.665	-4	16.297	5' N-5as	1116.672	-4	13.796	5' N-7s
1043.630	-4	16.543	3' N-8as	1198.930	-4	14.117	5' N-6s

1418.196	-4	17.119	5' N-3as	1217.849	-3	14.53	3' N-10s
1129.893	-4	17.119	3' N-7as	1358.460	-4	14.737	5' N-4s
1216.152	-4	17.63	3' N-6as	1440.724	-4	14.945	5' N-3s
1504.457	-4	17.729	5' N-2as	1602.991	-4	15.375	5' N-1s
1580.954	-4	17.94	5' N-1as	1351.206	-5	15.898	Sense
1108.478	-6	18.009	Antisense				
1454.931	-4	18.198	3' N-3as				
1531.187	-4	18.609	3' N-2as				
